# Etiology of Delayed Lactogenesis in Obesity

**DOI:** 10.3390/biomedicines13081848

**Published:** 2025-07-30

**Authors:** Gema Gomez-Casado, Natalia Saldaña-Garcia, Ernesto Gonzalez-Mesa, Almudena Ortega-Gomez

**Affiliations:** 1 Instituto de Investigación Biomédica de Málaga y Plataforma en Nanomedicina-IBIMA Plataforma BIONAND, 29590 Málaga, Spain; gema.gomez@ibima.eu (G.G.-C.); natalia@saldanagarcia.es (N.S.-G.); egonzalezmesa@uma.es (E.G.-M.); 2 Department of Surgical Specialties, Biochemistry and Immunology, Faculty of Medicine, Universidad de Málaga, 29071 Málaga, Spain; 3 Neonatology Department, Regional University Hospital of Málaga, 29011 Málaga, Spain; 4 CIBER Fisiopatologia Obesidad y Nutricion (CIBEROBN), Instituto de Salud Carlos III, 28029 Madrid, Spain

**Keywords:** obesity, lactogenesis, breastmilk, inflammation, hormone, circadian, mammary gland

## Abstract

Obesity is a multifactorial condition that influences metabolic, endocrine, inflammatory, circadian, and behavioral systems. These disruptions can adversely affect the initiation of lactogenesis II—the critical process marking the onset of copious milk secretion following childbirth. In mothers with obesity, prolonged inflammation within the mammary gland, a blunted hormonal response (notably of prolactin), altered progesterone and estrogen dynamics, high leptin levels, and misaligned circadian rhythms contribute significantly to delayed lactogenesis. In addition, mechanical difficulties and psychological factors further hinder effective breastfeeding. This report synthesizes evidence from human epidemiological studies and animal models that elucidate the diverse mechanisms linking maternal obesity to delayed lactogenesis. We review the role of obesity-associated inflammatory mediators in impairing mammary tissue remodeling, the endocrine aberrations that impair lactogenic signaling, the consequences of circadian disruption on hormonal rhythmicity, and the behavioral influences that challenge effective breastfeeding. Finally, we discuss the clinical implications of these findings and propose future research directions targeting endocrine modulation, anti-inflammatory therapy, circadian interventions, and enhanced lactation support strategies for mothers with obesity.

## 1. Introduction

Breastmilk is widely recognized as the optimal source of nutrition for newborns, providing a perfectly balanced combination of nutrients essential for healthy growth and development during the early stages of life. Beyond its unparalleled nutritional value, breastmilk offers powerful immunological benefits that help protect infants against a wide range of infectious and chronic diseases. These include gastrointestinal and respiratory infections, ear infections, and a reduced risk of conditions such as asthma, allergies, and obesity later in life [1].

Due to its numerous health advantages, the World Health Organization (WHO) strongly recommends exclusive breastfeeding for the first six months of a child’s life. During this period, infants should receive only breastmilk, without any additional food or drink—not even water. This practice supports optimal physical and cognitive development while strengthening the bond between mother and baby. After the initial six months, the WHO advises the gradual introduction of complementary foods—nutritious solid or semi-solid foods appropriate for the infant’s age—while continuing to breastfeed. This combined feeding approach is encouraged up to two years of age or beyond, as it continues to contribute significantly to the child’s health, development, and immune resilience during the critical early years of life [2].

Currently, around 650 million adults and approximately 340 million children and adolescents aged 5 to 19 are affected by obesity. The condition tends to be more common among women and older individuals compared to men and younger populations [3]. Obesity in adults significantly exacerbates the four most prevalent non-communicable diseases: cardiovascular disease, type 2 diabetes, various forms of cancer, and chronic respiratory diseases. Excess body fat contributes to increased inflammation, insulin resistance, and hormonal imbalances, all of which are underlying mechanisms that worsen these chronic conditions [4]. Obesity affects a significant proportion of women of reproductive age worldwide, with prevalence estimates ranging from 8% to over 40%, depending on geographic region and population studied. In the United States, nearly 40% of women aged 20–39 years are classified as obese [5], while European rates range from 8% to 26% among women aged 18–44 years [6]. These figures underscore the public health importance of understanding obesity’s implications for reproductive outcomes, including lactation.

A well-documented consequence of maternal obesity is delayed lactogenesis II (DLII), defined as the initiation of copious milk secretion occurring later than 72 h post-delivery [7]. The initiation of lactogenesis is not only essential for immediate neonatal nutrition but also plays a critical role in the long-term health of both mother and child. Mothers with obesity are at an increased risk of encountering complications such as gestational diabetes, hypertensive disorders, and cesarean delivery—all of which further compromise lactation outcomes [8].

Age has been described as an independent risk factor in delayed lactogenesis. Older mothers with obesity are more likely to experience delayed onset of copious milk secretion compared to their younger or normal-weight counterparts, highlighting an age-related vulnerability in lactation physiology [9,10]. Age-related changes in endocrine function, such as altered prolactin secretion and increased insulin resistance, may exacerbate the metabolic and inflammatory disturbances associated with obesity, further impairing milk production [11].

Extensive clinical research has consistently reported that maternal obesity is associated with a delayed onset of lactogenesis II (OL) (Table 1). However, the underlying mechanisms remain relatively underexplored. The etiology of DLII in mothers with obesity is multifactorial. Recent research implicates systemic and localized inflammation, endocrine and metabolic dysregulation, circadian misalignment, and behavioral barriers as interrelated mechanisms that delay the transition from colostrum production to the establishment of a copious milk supply. This report examines the current state of knowledge regarding the impact of obesity on lactogenesis with a particular emphasis on the inflammatory pathways activated by excess adiposity, the hormonal disturbances that result from both altered adipose endocrine function and the persistence of progesterone, the disruption of circadian hormonal rhythms, molecular mechanisms, and the behavioral challenges inherent in breastfeeding among mothers with obesity.

## 2. Inflammatory Mechanisms in Obesity-Related Delayed Lactogenesis

**Figure 1:**
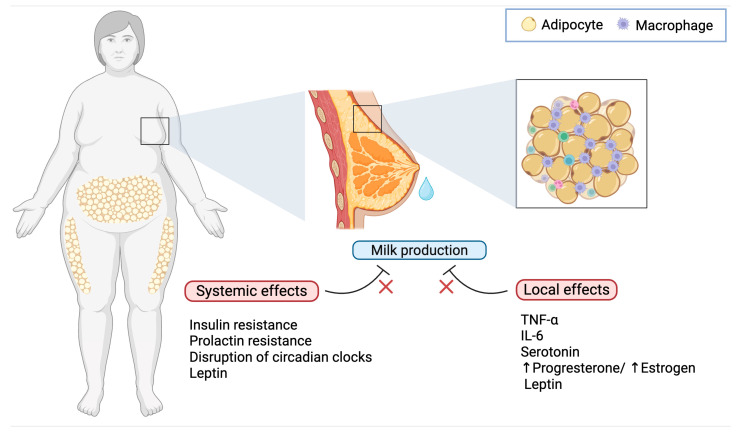
Effects of obesity on delayed lactogenesis II. Obesity interferes with lactation through both systemic and local mechanisms: while hormonal dysregulation, chronic inflammation, and circadian disruption impair lactogenesis at a systemic level, the mammary microenvironment in mothers with obesity is characterized by elevated levels of TNF-α and IL-6 from adipose tissue-infiltrating macrophages, increased serotonin, an imbalance in progesterone and estrogen, and elevated levels of leptin, all of which contribute to impaired milk production.

Obesity is characterized by an expansion of adipose tissue that becomes infiltrated by immune cells, notably macrophages, which secrete proinflammatory cytokines such as tumor necrosis factor-α (TNF-α) and interleukin-6 (IL-6) [30]. This systemic inflammatory state is further compounded by local inflammation within the mammary gland. The hypertrophy of mammary adipocytes leads to increased cell death and the formation of crown-like structures (CLS), where macrophages encircle necrotic adipocytes. Such localized inflammation interferes with the delicate remodeling of mammary tissue necessary for effective lactogenesis (Figure 1) [31]. Animal models have shown that inflammatory mediators not only impair the differentiation of mammary epithelial cells but also disrupt the expression of genes crucial for milk protein synthesis such as α-lactalbumin (milk protein gene important in regulation of milk volume) and β-casein (major milk protein, crucial for the development of the infant’s digestive and immune systems [32]. Furthermore, high-fat diet–induced obesity causes structural alterations in the mammary gland, including reduced alveolar development and parenchymal tissue, factors directly linked to delayed milk production [33].

Besides the overproduction of cytokines such as IL-6 and TNF-α within the inflamed mammary microenvironment, which has deleterious effects on mammary gland function, obesity is linked to increased synthesis of serotonin in the mammary gland by upregulating tryptophan hydroxylase 1 (TPH1). Elevated intramammary serotonin, via its receptor signaling, may mimic signals of involution and inhibit secretory function, thereby delaying the onset of lactogenesis II [32]. These findings from animal studies suggest that inflammatory processes triggered by obesity are a critical intermediary mechanism behind the perturbation of mammary gland structure and function, ultimately leading to delayed lactogenesis [33].

## 3. Hormonal and Endocrine Dysregulation

### 3.1. Blunted Prolactin Response

**Figure 2:**
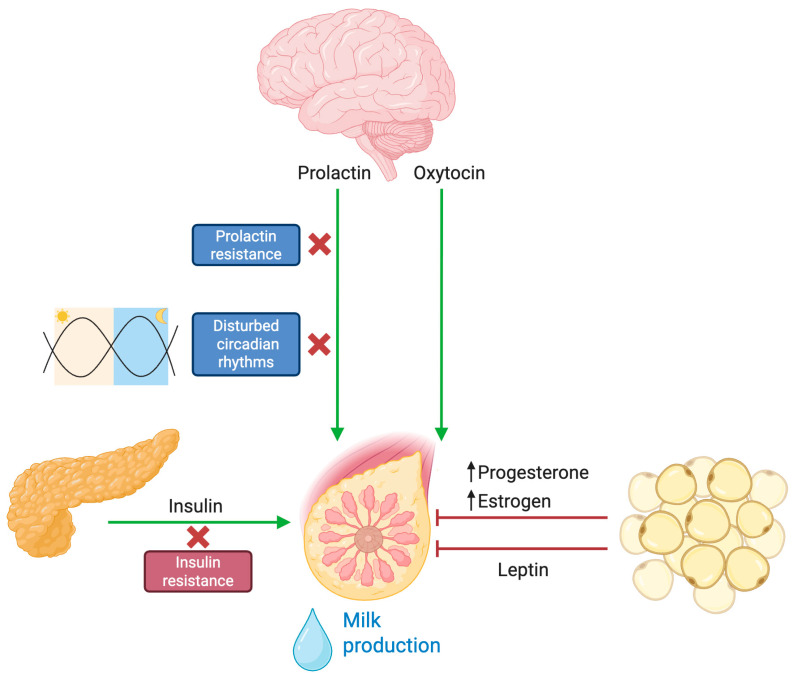
Hormonal and endocrine dysregulation of lactogenesis in obesity. Under physiological conditions, infant suckling activates sensory pathways that stimulate the hypothalamus to release prolactin and oxytocin. These hormones are essential for initiating milk synthesis (prolactin) and milk ejection (oxytocin) from the mammary glands. In the context of obesity, multiple disruptions impair this finely tuned process. Prolactin signaling and insulin sensitivity are reduced, hindering the initiation and maintenance of adequate milk output. Additionally, altered circadian rhythms associated with obesity lead to hormonal imbalances that negatively affect lactation. Elevated leptin levels—commonly found in obesity—have been implicated in limiting the mammary gland’s ability to sustain robust lactation. Furthermore, excess adipose tissue acts as a hormonal reservoir for progesterone and estrogen, delaying the necessary postpartum decline in these hormones that is crucial for a successful onset of milk secretion.

Prolactin is the key hormone that drives lactogenesis. In a normally functioning lactogenic system, the act of infant suckling elicits a rapid and robust release of prolactin, which in turn stimulates milk synthesis. However, multiple studies have indicated that mothers with obesity exhibit a provoked lower prolactin response to suckling in the early postpartum period [34]. Both clinical observations and animal models have provided evidence that obesity induces central and peripheral prolactin resistance. In obese murine models, high basal levels of phosphorylated STAT5 (a downstream mediator of prolactin receptor signaling) are noted, yet the acute stimulatory response to prolactin is markedly attenuated [33]. This hormonal insensitivity compromises the induction of lactogenesis II, resulting in a delay in the onset of copious milk production (Figure 2).

### 3.2. Progesterone Dynamics and Local Estrogen Production

A critical trigger for lactogenesis II is the gradual withdrawal of progesterone following parturition. In normal physiology, the removal of the placental source of progesterone relieves its inhibitory effect on lactogenesis. In women with obesity, however, excess adipose tissue serves as a reservoir for progesterone, resulting in prolonged exposure to the hormone after delivery [34,35,36]. This persistence of progesterone delays the activation of the secretory apparatus within the mammary gland. In addition to progesterone issues, obesity increases the aromatization of androgens into estrogen within adipose tissue. The consequent rise in local estrogen is believed to antagonize prolactin signaling and downregulate genes necessary for milk synthesis [34,37]. These endocrine alterations—marked by both sustained progesterone levels and enhanced estrogenic activity—create an unfavorable hormonal milieu that postpones lactogenic transition, thereby delaying the onset of copious milk secretion [38].

### 3.3. Insulin Resistance

Insulin is recognized as an important regulator of mammary epithelial cell function and lactation. In women with obesity, the state of insulin resistance and hyperinsulinemia disrupts the normal metabolic reprogramming necessary for milk synthesis. This metabolic impairment hinders the delivery of glucose and other substrates that are vital for the synthetic machinery of milk production [39]. Moreover, the increased prevalence of gestational diabetes among mothers with obesity further exacerbates these impairments by altering insulin signaling cascades within the mammary tissue [40]. Consequently, the metabolic dysregulation intrinsic to obesity results in reduced availability of essential nutrients for lactogenesis, further compounding the delay in milk secretion.

### 3.4. Lepin and the Control of Lactogensis

Leptin, a hormone primarily known for its role in energy homeostasis, has emerged as a key factor in mammary gland physiology and lactogenesis. Adipose tissue is the main source of leptin production, and this includes adipose deposits within the mammary gland. During pregnancy, leptin acts similarly to a growth hormone, with maternal levels declining after birth [41]. Experimental evidence in mouse models indicates that while leptin mRNA is clearly present in the mammary epithelium, its production is downregulated during lactogenesis, suggesting that high local leptin levels might be counterproductive for maximal milk protein synthesis [42]. Leptin hinders milk ejection in part by directly targeting myoepithelial cells, which contract in response to oxytocin to help expel milk [33]. Women with obesity tend to have elevated leptin levels and may exhibit leptin resistance. Studies have shown that leptin concentrations remain significantly higher in obese women during lactation, specifically at 48 h and 7 days postpartum [34]. Consequently, high maternal leptin levels have been associated with delayed onset of lactogenesis II and attenuated milk ejection, likely because of leptin’s inhibitory effects on oxytocin-induced myometrial contractions [38].

## 4. Circadian Disruption and Its Impact on Lactogenesis

Circadian rhythms, regulated by the central clock in the suprachiasmatic nucleus of the hypothalamus, govern the periodic secretion of hormones and maintain metabolic homeostasis. In the context of lactation, these biological rhythms ensure that hormones such as prolactin, cortisol, and insulin are released in a coordinated manner to optimize milk production [43]. Peripheral clocks within the mammary gland further modulate local gene expression, aligning the gland’s functional activity with the body’s overall circadian schedule.

Studies in mice show that disrupting circadian clock genes, particularly through the Clock-Δ19 mutation, impairs lactation competence without significantly affecting fetal development [44]. Mutant dams exhibited abnormal nursing behavior, disrupted prolactin rhythms, reduced mammary gland development, and lower offspring survival. These effects were linked to peripheral rather than central clock dysfunction. Further experiments revealed that reducing CLOCK protein in mammary cells decreased the expression of genes involved in milk synthesis, indicating that proper circadian function is crucial for successful lactation, mammary differentiation, and milk production [45,46].

Obesity has been shown to disrupt normal circadian rhythms, primarily through behavioral factors such as irregular sleep patterns, exposure to artificial light at night, and altered meal timings. Such circadian misalignment blunts the nocturnal rise in melatonin and disturbs cortisol rhythms, which in turn have downstream effects on metabolic and reproductive hormone secretion [47]. These disrupted hormonal patterns hinder the normal pulsatile release of prolactin in response to suckling, thereby delaying lactogenesis [43]. The difficulty in synchronizing the timing of lactogenic signals due to circadian disturbance may add a layer of complexity to the endocrine challenges faced by mothers with obesity. While further studies in humans are needed to characterize the potential circadian rhythm disruption caused by obesity in lactating women, and current statements remain conjectural, this aspect could represent another piece of the puzzle in the etiology of delayed lactogenesis.

## 5. Mechanisms Underlying Impaired Lactogenesis in Obesity

**Figure 3:**
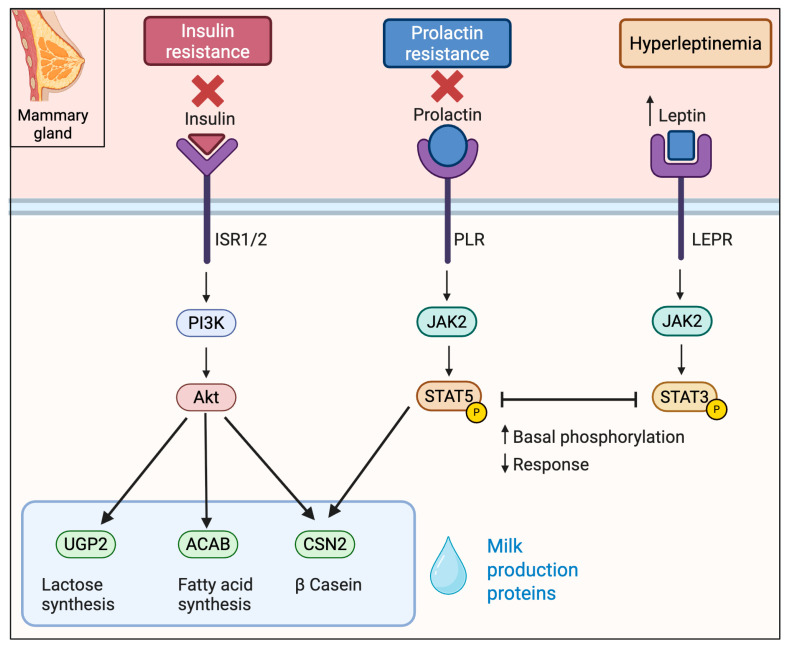
Key signaling cascades in mammary gland affected by obesity in lactogenesis. Obesity promotes insulin and prolactin resistance and is characterized by elevated leptin levels. Insulin signaling through the PI3K–AKT pathway, essential for milk protein synthesis, is impaired. Simultaneously, high basal phosphorylation of STAT5 downstream of the prolactin receptor leads to a blunted prolactin response. Elevated leptin engages the JAK2–STAT3 pathway preventing further STAT5 activation and contributing to delayed lactogenesis.

Obesity-induced insulin resistance blunts anabolic signaling of the mammary gland. Under normal lactation, insulin acting via Insulin Receptor (INSR)—Insulin Receptor Substrate IRS1/2- Phosphoinositide 3-Kinase (PI3K)—Protein Kinase B (AKT) drives alveolar differentiation and milk synthesis, while prolactin signals via Prolactin Receptor (PRLR)- Janus Kinase 2 (JAK2)—(STAT5) to induce milk-protein genes (e.g., *CSN2* for β-casein) [48,49]. In obese, insulin-resistant women, mammary insulin signaling is suppressed, e.g., *IRS2* mRNA was ~2.3-fold lower in lactating obese vs. lean women [50]. Correspondingly, downstream anabolic genes are downregulated as follows: lactogenic genes *ACACB* (fatty acid synthesis), *UGP2* (lactose synthesis), and β-casein (*CSN2*) are 3.1, 2.7, and 2.1 fold decreased. Notably, genes in the PRLR/JAK2/STAT5 pathway were unchanged, suggesting the defect is functional. Indeed, animal studies show obese dams have high basal mammary pSTAT5 but fail to respond to prolactin (true prolactin resistance) [33]. Thus, obesity creates an “insulin-resistant” gene signature in mammary without gross loss of PRL receptor, implicating downstream or cross-talk inhibition of STAT5 (Figure 3).

Dysregulated adipose-derived signals in obesity impair lactogenic signaling via several pathways. In obesity, leptin is chronically elevated. Mammary epithelial cells express the long leptin receptor (LEPR), especially on myoepithelial cells. High leptin signaling activates JAK2-STAT3 pathways that antagonize prolactin/STAT5. For example, in obese mice leptin produced high basal mammary pSTAT5 and prevented further STAT5 activation by prolactin [33]. In humans, when maternal leptin levels are elevated, there is a diminished PRL output—obese mothers have a blunted prolactin response to suckling and lower milk volume with higher leptin [51] (Figure 3).

In obesity, skeletal muscle becomes insulin resistant with decreased IRS1 and Glucose Transporter protein type 4 (GLUT4), contributing to systemic hyperinsulinemia [52]. Muscle also secretes myokines, such as IL-6, which have been implicated in contributing to chronic JAK-STAT3-mediated insulin resistance under obese conditions [53].

Hepatic insulin resistance in obesity leads to elevated gluconeogenesis and dysregulated insulin growth factor/growth hormone (IGF/GH) signaling [54]. The liver is the main source of IGF-1 and binding proteins (IGFBPs) that support mammary development [55]. 

In obesity, altered IGF-1/IGFBP levels may impair IGF-1R-IRS/PI3K signaling in mammary tissue, compromising the production of milk [56,57].

## 6. Behavioral Influences and Clinical Implications

### 6.1. Mechanical and Ergonomic Challenges

In addition to the biochemical impairments, mothers with obesity frequently encounter behavioral and mechanical challenges during breastfeeding. Physical attributes associated with obesity, such as increased breast size and postpartum edema, can interfere with proper infant latch and positioning [58]. These mechanical difficulties can lead to ineffective milk removal, which prevents the necessary feedback loop that stimulates prolactin release. Research indicates that when mothers experience poor latch or insufficient suction, the reduced stimulation further blunts the hormonal signals required for lactogenesis, thereby exacerbating delayed milk production [59].

### 6.2. Phycological and Sociocultural Barriers

Obesity is also frequently accompanied by psychological stresses, including lower self-efficacy and body image concerns. These factors can have a profound impact on a mother’s confidence and willingness to initiate and continue breastfeeding [60]. The stigma associated with obesity may deter some mothers from seeking the specialized lactation support they require, further deepening the challenges associated with delayed lactogenesis. In addition, the psychological stress imposed by a negative body image may induce further endocrine disturbances, including elevated cortisol levels, which are known to interfere with lactogenic hormonal cascades and breastmilk composition [61,62].

### 6.3. Clinical Practice and Intervention Strategies

The clinical implications of obesity-induced delayed lactogenesis are significant. Mothers with obesity are more prone to obstetric complications such as cesarean delivery, gestational diabetes, and hypertensive disorders, all of which independently contribute to delays in lactogenesis [8,63]. In a clinical setting, early identification of at-risk mothers is essential. Interventions such as proactive lactation consultation, specialized breastfeeding education, and the utilization of mechanical aids to improve infant latching can mitigate some of the challenges posed by obesity [64]. The incorporation of multidisciplinary care, including endocrinologists, nutritionists, and mental health professionals, is also critical in addressing both the physiological and behavioral dimensions of delayed lactogenesis.

## 7. Discussion

The evidence presented in this report underscores that delayed lactogenesis in mothers with obesity is the product of a complex interplay among inflammatory, hormonal, circadian, and behavioral factors. Inflammation provoked by excess adipose tissue compromises mammary gland architecture and function by interfering with epithelial cell differentiation and reducing the expression of key milk synthesis genes [32,33]. At the same time, obesity dampens the prolactin response to suckling—a pivotal hormonal signal for lactogenesis—which is compounded by prolonged progesterone exposure and increased local estrogen production due to enhanced aromatization in adipose tissue [35,65]. Metabolic disturbances, including insulin resistance commonly seen in mothers with obesity, contribute further by limiting the availability of energy substrates necessary for milk production. In this hormonal balance, elevated leptin levels contribute to reduced milk secretion by counteracting the effects of oxytocin. Circadian misalignment appears as another significant factor; the disruption of the normal hormonal oscillations—particularly that of prolactin—impairs the synchronized activation of lactogenic processes [66]. Furthermore, behavioral influences—ranging from mechanical challenges in latch and infant positioning to psychosocial stress and lower self-esteem—create a self-reinforcing cycle that exacerbates lactational insufficiency. Ineffective breastfeeding practices reduce the suckling-induced prolactin surge, thereby further delaying milk production [59]. Importantly, these diverse factors do not operate in isolation; rather, they interact synergistically. For instance, inflammation may influence circadian clock gene expression, while endocrine imbalances may heighten psychological stress.

Despite the evidence identified between obesity and delayed lactogenesis, it is important to consider the concept of metabolically healthy obesity. A substantial proportion of mothers with obesity are able to initiate and sustain successful breastfeeding without requiring additional support. This highlights the heterogeneity within the population of women with obesity and suggests that not all individuals are equally affected by the metabolic and hormonal disruptions often linked to delayed lactogenesis.

While the primary factors contributing to the association between obesity and delayed lactogenesis—such as metabolic disturbances, hormonal imbalances, inflammation, and behavioral challenges—have been increasingly elucidated, further research is essential to fully complete the picture. Notably, there remains significant variability in how delayed lactogenesis is defined and measured across studies, as highlighted in Table 1, underscoring the urgent need for standardized diagnostic criteria and assessment practices. Moreover, current evidence often fails to distinguish between metabolically healthy obesity and obesity accompanied by metabolic dysfunction. The physiological differences between these phenotypes, and how they might differentially impact lactogenesis, remain largely unknown and represent a critical gap in our understanding that warrants future investigation.

## 8. Future Directions

Given the multifaceted nature of obesity-induced delayed lactogenesis, future research should adopt a multidisciplinary approach that encompasses molecular, physiological, and behavioral dimensions. Key areas for future investigation should include longitudinal studies that follow mothers with obesity from the prenatal period through postpartum lactation to fully characterize the interplay between metabolic, inflammatory, endocrine, and behavioral factors. By addressing these research directions, it will be possible to develop targeted therapeutic strategies that mitigate the adverse impacts of obesity on lactogenesis. Such approaches are essential not only for improving immediate breastfeeding outcomes but also for interrupting the intergenerational cycle of obesity and metabolic dysfunction.

**Figure 4:**
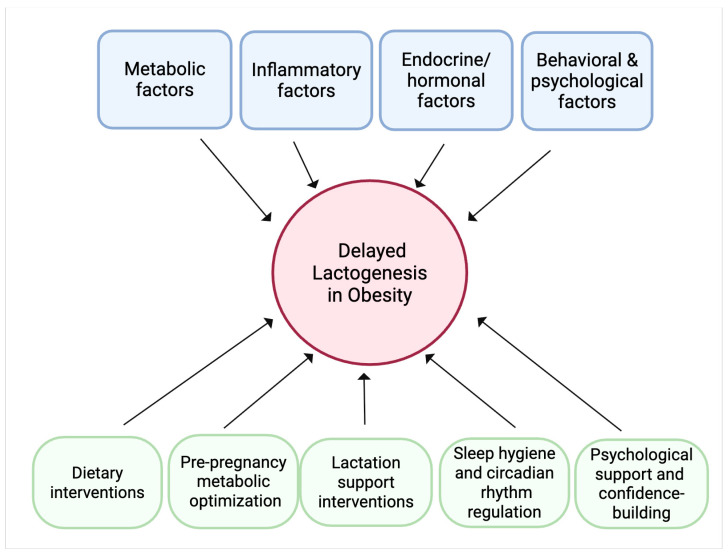
Conceptual framework of the conclusions and future directions. Obesity can lead to delayed lactogenesis through the interplay of metabolic, inflammatory, endocrine/hormonal, and behavioral and psychological factors. Potential therapeutic strategies to mitigate these effects encompass dietary modifications rich in anti-inflammatory nutrients, metabolic optimization before and during pregnancy, hormonal therapies or lifestyle changes to support endocrine balance, sleep hygiene and circadian rhythm regulation, specialized lactation support, and psychological interventions to improve maternal confidence and reduce stress.

Addressing delayed lactogenesis in mothers with obesity requires a comprehensive, multidisciplinary approach that targets the multifactorial pathways implicated in this condition. Therapeutic strategies should focus on mitigating systemic and local inflammation through dietary modifications rich in anti-inflammatory nutrients. Optimizing metabolic health prior to and during pregnancy—through interventions aimed at improving insulin sensitivity and controlling gestational diabetes—can help restore the anabolic signaling pathways essential for mammary gland function. Hormonal imbalances, including prolonged progesterone exposure and elevated leptin levels, might be managed through carefully timed hormonal therapies or lifestyle changes that support endocrine regulation. Behavioral and mechanical challenges can be alleviated by providing specialized lactation support, including expert breastfeeding education, ergonomic guidance to improve infant latch, and the use of assistive devices. Additionally, circadian rhythm stabilization through sleep hygiene interventions and controlled light exposure may enhance the coordinated release of lactogenic hormones such as prolactin and cortisol. Psychological support is equally critical to address body image concerns and reduce stress-related endocrine disruption, thereby improving maternal confidence and breastfeeding self-efficacy. Together, these strategies, tailored to individual needs, hold promise for overcoming the barriers posed by obesity and promoting timely onset of copious milk production, ultimately improving breastfeeding success and maternal-infant health outcomes (Figure 4).

## 9. Conclusions

Obesity is associated with potential challenges in initiating lactogenesis due to multiple interconnected pathways. The chronic, low-grade inflammatory state associated with obesity disrupts the normal remodeling of the mammary gland, while hormonal dysregulation—manifested as a blunted prolactin response, prolonged progesterone presence, increased local estrogen production, and metabolic disturbances due to insulin resistance—further impairs the onset of copious milk production. In addition, obesity-related circadian misalignment contributes to the disruption of the timing of lactogenic hormonal release, and mechanical as well as psychosocial barriers reduce effective breastfeeding practices. Together, these factors culminate in a significant delay in lactogenesis II, undermining successful breastfeeding outcomes and potentially influencing long-term metabolic and developmental trajectories in the offspring and the mother.

## Figures and Tables

**Table 1:** Overview of studies investigating obesity in relation to breastfeeding.

Authors	Type of Study	Country	Groups of Study	N	Conclusions	Year	Data Collection	Comparison	Findings (OR, AOR, HR, Mean, %)	CI/SD	*p*-Value
[12]	Cross-sectional study	Mexico	Normal Weight	863	Higher BMI was associated with greater body image dissatisfaction, which in turn was associated with lower likelihood of currently breastfeeding or having breastfed.	2018	Survey (AOR of the associations between breastfeeding and maternal weight status)				
			Overweight	926				Overweight (vs. normal weight)	1.01	95%CI: 0.62–1.62	n.s.
			Obesity	633				Obese (vs. normal weight)	0.60	0.38–0.96	<0.05
			Total	2422							
[13]	Randomized trial	UK	Obesity I (BMI 30.0–34.9 kg/m^2^)	354	The length of exclusive breastfeeding tended to be shorter with higher BMI. Breastfeeding-related changes in body size and shape were not noticeable among women of Black ethnicity	2024	Survey (difference in mean duration of breastfeeding in days)	Obesity II vs. I	−15.8	95%CI: −28.5, −3.1	<0.05
			Obesity II (BMI 35.0–39.9 kg/m^2^)	225							
			Obesity III (BMI ≥ 40.0 kg/m^2^)	136				Obesity III vs. I	−16.7	−32.0, −1.35	<0.05
			Total	715							
[14]	Retrospective cohort study	USA	Gestational weight gain over recommended	117	In women with class III obesity, excessive gestational weight gain did not affect the rates of exclusive breastfeeding at discharge or during the postpartum visit	2022	Survey (% exclusive breastfeeding at discharge in obesity class III)	At discharge (weight gain as vs. over recommended)	66.7 vs. 70.9		n.s.
			Gestational weight gain as recommended	177				At postpartum visit (weight gain as vs. over recommended)	40.1 vs. 34.2		n.s.
			Total	294							
[15]	Cross-sectional study	Australia	Underweight	372	Mothers with obesity breastfed for a significantly shorter duration compared to mothers without obesity. This difference persisted even after accounting for factors like maternal smoking, age, and other sociodemographic variables	2000	Survey (AOR of stopping breastfeeding according to BMI)	<25 kg/m^2^	1		
			Normal weight	1184							
			Overweight	490				25–30 kg/m^2^	1.15	95%CI: 1.01–1.31	<0.05
			Obesity	254				>30 kg/m^2^	1.36	1.15–1.61	<0.05
			Not known/stated	312							
			Total	2612							
[16]	Cross-sectional study	Australia	Normal weight	1567	Overweight women had similar breastfeeding cessation rates in the first week and up to 6 months, whereas obese women were more likely to stop breastfeeding in the first week than later	2008	Survey (AOR of ceased lactation at first week vs. 6 months)	Normal weight	1		
			Overweight	890				Overweight	1.15	95%CI: 1.01–1.31	<0.05
			Obesity	618				Obesity	1.36	1.15–1.61	<0.05
			Total	3075							
[17]	Cross-sectional study	Australia	Normal Weight	108	High maternal BMI has a significant negative effect on breastfeeding success during the early postnatal period	2021	Survey (OR of breastfeeding at 8 weeks)	Overweight vs. normal BMI	1.48	95%CI: 0.79–2.78	n.s.
			Overweight	103							
			Obesity	76				Obesity vs. normal BMI	3.97	2.08–7.55	<0.05
			Morbid Obesity	10							
			Total	297							
[18]	Case–control study	Germany	Normal Weight	70	A smaller proportion of mothers with obesity breastfed compared to normal-weight mothers, and their breastfeeding duration was significantly shorter-even after accounting for education and family income	2016	Survey (mean breastfeeding obesity vs. normal weight)	Breastfeeding practice (months)	5.8 vs. 8.8	SD: 4.5 vs. 5	<0.05
			Obesity	80				Exclusive breastfeeding duration (months)	3,9 vs. 5,1	2.5 vs. 2.1	<0.05
			Total	150							
[19]	Cross-sectional study	USA	Never obesity	110	Individuals who developed excess adiposity before or during puberty were 1.6 times more likely to have exclusive breastfeeding failure (less than 6 months) compared to those whose excess adiposity began after puberty	2019	Survey (exclusive breastfeeding < 6 moths)	Excess adiposity onset before or during puberty vs. after puberty	1.58		<0.05
			Obesity after puberty	991							
			Obesity before/during puberty	469							
			Total	1570							
[20]	Prospective cohort study	China	Underweight	619	Women with higher gestational weight gain throughout pregnancy are more likely to suffer from delayed onset of lactation in Chinese population	2019	Survey (AOR of maternal perception of noticeable breast fullness after 72 h postpartum among gestational weight gain quartiles)	Quartile 1	1		
			Normal Weight	2296				Quartile 2	1.20	95%CI: 0.91–1.57	n.s.
			Overweight	367				Quartile 3	1.47	1.13–1.92	<0.05
			Total	3282				Quartile 4	1.42	1.08–1.86	<0.05
[21]	Cross-sectional study	USA	Normal/underweight	854	Two practices: holding their babies skin-to-skin immediately after birth and being encouraged to breastfeed on demand were more strongly linked to exclusive breastfeeding in mothers with obesity than in other mothers. However, mothers with obesity reported practicing skin-to-skin contact significantly less frequently than others	2019	Survey (AOR of exclusive breastfeeding at 1 week and 3 months on skin-to-skin practice)	Normal/underweight	1.07 and 0.87	95%CI: 0.76–1.51 and 0.62–1.21	n.s.
			Overweight	378				Overweight	1.62 and 1.86	0.97–2.71 and 1.12–3.09	<0.05
			Obesity	274				Obesity	3.18 and 1.66	1.73–5.85 and 0.94–2.94	≤0.01
			Total	1506			Survey (AOR of exclusive breastfeeding at 1 week and 3 months on being encouraged to breasted on demand practice)	Normal/underweight	1.16 and 1.08	95%CI: 0.80–1.69 and 0.75–1.56	n.s.
								Overweight	1.00 and 0.69	0.57–1.77 and 0.39–1.20	n.s.
								Obesity	2.32 and 2.29	1.09–4.56 and 1.10–4.78	≤0.01
[22]	Randomized trial	Denmark	Low BMI (<27 kg/m2)	871	Mothers with high postpartum BMI were more likely to stop exclusive breastfeeding early and often had socio-demographic, psychosocial, perinatal, and behavioral factors that increase the risk of early breastfeeding cessation compared to other mothers	2013	Survey (HR of breastfeeding cessation before 17 weeks postpartum)	Low BMI	1		
			Moderate (27 ≤ BMI < 32 kg/m2)	344				Moderate BMI	1.16	95%CI: 0.84–1.24	<0.05
			High (≥32 BMI kg/m2)	160				High BMI	1.21	0.93–1.57	<0.05
			Total	1375							
[23]	Randomized trial	USA (African American Women)	Home-based parenting support	59	African American women with overweight or obesity who took part in a home-based educational program breastfed at rates higher than the national average for black women	2018	Survey (RR of breastfeeding after receiving home-based parenting support with or without)	With breastfeeding education vs. without	1.05	95%CI: 0.86–1.28	<0.05
			Home-based parenting support with additional content on breastfeeding	59							
			Total	118							
[24]	Cross-sectional study	USA	Underweight	213,108	Overweight and obese non-Hispanic white women and obese non-Hispanic black women were more likely to not start breastfeeding. No link was found for Hispanic or other racial groups.	2015	Survey (AOR of breastfeeding cessation according to BMI depending in white, non-Hispanic women)	Underweight	1.18	95%CI: 0.99–1.40	n.s.
			Normal Weight	2,477,498				Normal Weight	1		
			Overweight (	1,186,841				Overweight	1.17	1.07–1.29	<0.05
			Obesity Total	1,041,886				Obesity	1.25	1.14–1.37	<0.05
				4,919,333			Survey (COR of breastfeeding cessation according to pre-pregnancy BMI depending in Hispanic women=	Underweight	1.36	95%CI: 0.77–2.39	n.s
								Normal Weight			
								Overweight	1.00	0.77–1.30	n.s
								Obesity	1.32	1.02–1.71	<0.05
[10]	Case–control study	USA	Timely OL	588	Maternal obesity, insulin therapy, and inadequate breastfeeding support in the hospital were major risk factors for delayed lactation onset.	2014	Survey (AOR of maternal perception of the onset of lactation)	Overweight	1.26	95%CI: 0.85- 1.85	n.s
			Delayed OLs	295				Obesity	1.56	1.07–2.29	<0.05
			Total	883							
[9]	Prospective cohort study	USA	Timing OL non-obesity	102	After accounting for prenatal feeding plans, having overweight or obesity were key factors linked to delayed onset of lactation	2010	Survey (AOR of reported delayed lactogenesis according to BMI)	Overweight vs. Normal Weight	1.84	95%CI: 1.07–3.16	<0.05
			Timing OL obesity	34				Obesity vs. Normal Weight	2.21	1.24–3.94	<0.05
			Delayed OL non-obesity	49							
			Delayed OL-obesity	33							
			Total	218							
[25]	Retrospective cohort study	Japan	BMI at discharge:		Compared to women with normal weight, those with obesity were much less likely to successfully start exclusive breastfeeding. Gaining more weight during pregnancy also slightly reduced the chances of exclusive breastfeeding initiation	2019	Medical records (AOR of successful exclusive breastfeeding at discharge)				
			Underweight	404				Underweight	1.03	95%CI: 0.87–1.23	n.s.
			Normal Weight	4.443				Normal Weight	1.00		
			Overweight	224				Overweight	0.70	0.50–0.99	<0.05
			Obesity	54				Obesity	0.27	0.15–0.51	<0.001
			BMI at 1 month after delivery:				(AOR of successful exclusive breastfeeding 1 month after delivery)				
			Underweight	1.236				Underweight	0.99	95%CI: 0.85–1.16	n.s.
			Normal Weight	3.900				Normal Weight	1.00		
			Overweight	164				Overweight	0.81	0.56–1.16	n.s.
			Obesity	48				Obesity	0.29	0.16–0.53	<0.001
			Total	10.473							
[26]	Prospective cohort study	Australia	Normal Weight	1479	Pre-pregnancy body mass index is linked to shorter breastfeeding duration, with overweight and obesity mothers tending to breastfeed for less time than normal-weight mothers, regardless of their socioeconomic or demographic background	2006	Survey (HR of breastfeeding duration with overweight or obesity)	Pre-pregnancy overweight or obesity vs. normal weight	1.18	95%CI: 1.05–1.34	<0.05
			Overweight	211							
			Obesity	113							
			Total	1803							
[27]	Retrospective cohort study	Canada	Normal weight	5685	Women with obesity were less likely to breastfeed compared to those with normal weight, and obesity independently increased the risk of not breastfeeding at hospital discharge	2016	Database (AOR of breastfeeding at discharge)	Obesity vs. normal weight	0.63	95%CI: 0.55–0.71	<0.001
			Obesity	3343							
			Total	9028							
[28]	Prospective cohort study	China	Underweight	605	In Chinese women, pre-pregnancy obesity raises the risk of delayed onset of milk production and early discontinuation of breastfeeding	2017	Survey (RR of delayed of maternal perception of breast fullness)	Underweight	0.84	95%CI: 0.58–1.22	n.s.
			Normal Weight	2209				Overweight	1.38	0.90–2.12	n.s.
			Overweight	300				Obesity	1.89	1.04–3.4	<0.05
			Obesity	82							
			Total	3196							
[29]	Retrospective cohort study	Canada	Underweight	337	Maternal obesity was associated with a two-fold rate of non-initiation of breastfeeding.	2015	Survey (RR of non-initiation of breastfeeding)	Underweight	0.97	95%CI: 0.75–1.25	n.s.
			Normal weight	4105				Normal weight	1.00		
			Overweight	1317				Overweight	1.07	0.92–1.24	n.s.
			Obesity	833				Obesity	1.22	1.04–1.42	<0.05
			Total	6592							

SD, Standard Deviation; CI, Confidence Interval; OR, Odds Ratio, AOR, Adjusted Odds Ratio; HR, Hazard Ratio; RR, Relative Risk; BMI, Body Mass Index; OL, Onset of Lactogenesis; n.s., non-significant.

## Abbreviations

The following abbreviations are used in this manuscript:

DLII	Delayed Lactogenesis II
OL	Onset of Lactogenesis
TNF-α	Tumor Necrosis Factor-alpha
IL-6	Interleukin-6
CLS	Crown-Like Structures
TPH1	Tryptophan Hydroxylase 1
STAT5	Signal Transducer and Activator of Transcription 5
CLOCK	Circadian Locomotor Output Cycles Kaput
INSR	Insulin Receptor
IRS1/2	Insulin Receptor Substrate
PI3K	Phosphoinositide 3-Kinase
JAK2	Janus Kinase 2
AKT	Protein Kinase B
LEPR	Leptin Receptor
GLUT4	Glucose Transporter Protein Type 4
IGF	Insulin Growth Factor
IGFBP	Insulin Growth Factor Binding Protein
